# Adipose Tissue Stress-Related Changes in Mice and Humans with HFpEF

**DOI:** 10.1016/j.jacbts.2025.04.019

**Published:** 2025-08-25

**Authors:** Lisandro Maya-Ramos, Philipp E. Scherer

**Affiliations:** a Touchstone Diabetes Center, University of Texas Southwestern Medical Center, Dallas, Texas, USA; b Cardiology Division, Department of Internal Medicine, University of Texas Southwestern Medical Center, Dallas, Texas, USA

**Keywords:** browning, fibrosis, HFpEF, obesity, white adipose tissue

**Figure:**
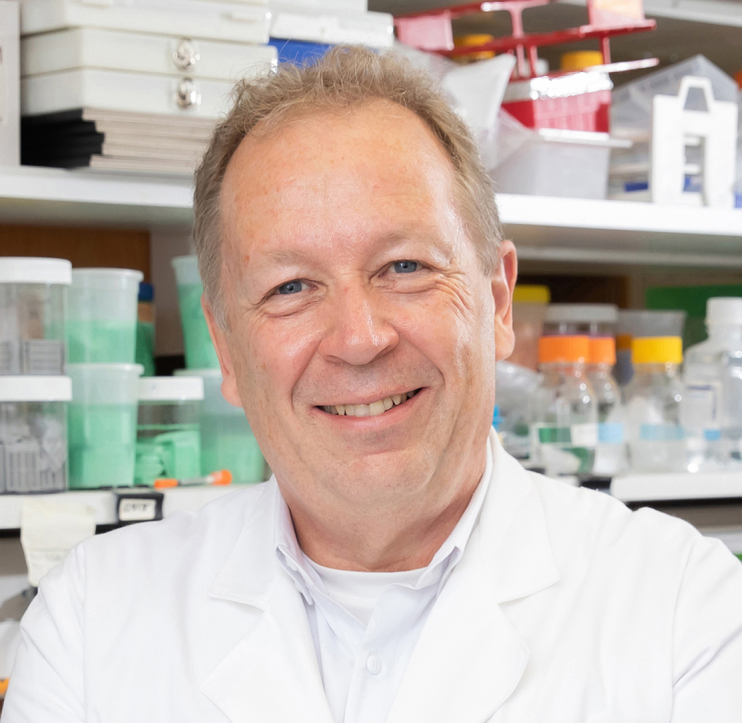

Heart failure with preserved ejection fraction (HFpEF) has emerged as a significant public health problem, now accounting for nearly one-half of heart failure–related hospital admissions.^1^ HFpEF is a complex and heterogeneous condition often associated with multiple comorbidities, including obesity. Over the last 5 decades, the global obesity rates have nearly tripled.^2^ In the United States, approximately 42% of adults are classified as obese. Obesity is not only a strong risk factor for HFpEF, but has also been recognized as a key pathophysiological driver of HFpEF.^2^^,^^3^ The advent of weight loss drugs, particularly GLP-1 receptor agonists, has placed adipose tissue in the spotlight as a potential therapeutic target for HFpEF. Notably, the STEP-HFpEF (Semaglutide Treatment Effect in People with obesity and HFpEF) trial demonstrated that patients with HFpEF and obesity treated with semaglutide experienced significant weight loss and a notable improvement in overall health status.^4^ As a result, investigating adipose tissue dynamics in the context of HFpEF is highly pertinent to addressing the HFpEF-obesity syndemic.

In this issue of *JACC: Basic to Translational Science*, Valero-Muñoz et al^5^ investigated the changes in white adipose tissue (WAT) in patients with HFpEF and in a preclinical mouse model of the condition. Previous studies have shown changes in WAT, including browning, during periods of metabolic stress, including in HFpEF.^6^ Building on this, Valero-Muñoz et al^5^ further assessed changes in WAT in a mouse model of HFpEF and obese sham controls. Subcutaneous WAT from HFpEF mice exhibited smaller adipocyte size and increased expression of the brown adipose tissue marker Ucp1. However, other browning markers, such as *cidea* and *eva*, did not show similar up-regulation. Additionally, HFpEF mice demonstrated reduced expression of classical white fat markers—*adiponectin*, *leptin*, and *resistin—*relative to obese sham animals. These results suggest that WAT shifts toward a brown phenotype, or it may simply reflect a degree of reduced overall adipogenic drive under these conditions.

Endothelial cell markers (*Cdh5*, *Pecam1*, *Vegfa*, and *Hif1a*) were also down-regulated in HFpEF mice, indicating a more hypoxic WAT environment, which may contribute to inflammation and fibrosis. Notably, inflammatory markers (*Il1b*, *Il6*) were altered only in visceral WAT, not in subcutaneous WAT. Similarly, expression of the fibrosis-associated gene *Col1a* was increased in visceral WAT but decreased in subcutaneous WAT in HFpEF mice compared with control subjects. Collectively, these findings suggest a stress response in WAT, particularly in the visceral fat depot, under HFpEF conditions.

To evaluate the relevance of their findings in humans, Valero-Muñoz et al^5^ analyzed adipose tissue biopsy samples from patients with acutely decompensated HFpEF, the same patients 1 month postdischarge (stable HFpEF), and a cohort of obese control individuals. Several gene expression differences emerged when comparing HFpEF patients (both acute and stable) to obese control subjects, some of which differed from those observed in the preclinical model. Key differences were as follows: 1) increased *UCP1* and *TNFA* transcript levels in acutely decompensated HFpEF patients compared with control subjects; 2) elevated *RETN* transcript levels in stable HFpEF patients; 3) decreased *COL1A*, *COL3A*, and *COL6A* expression in both acute and stable HFpEF patients; and 4) no significant changes in endothelial markers between HFpEF patients and control subjects.

These differences may be influenced by baseline disparities between HFpEF and control groups. As noted by the authors, control participants were, on average, more than 20 years younger than HFpEF patients (37 years vs 62.5 years). Moreover, the HFpEF cohort had a higher prevalence of comorbidities—including hypertension (100% vs 0%), atrial fibrillation (9% vs 0%), chronic kidney disease (45.5% vs 0%), and cigarette use (45.5% vs 0%)—which were absent in the control group. Such comorbid conditions, along with aging itself, are known to contribute to adipose tissue dysfunction, characterized by impaired adipogenesis, insulin resistance, dysregulated adipokine production, and inflammation.^7^

Further, in both clinical and preclinical contexts of CKD, changes in energy expenditure, mitochondrial activity, and WAT browning have been observed.^8^^,^^9^ Likewise, nicotine exposure has been shown to promote WAT browning, with smokers exhibiting higher *UCP1* levels than nonsmokers.^10^ These confounding factors must be carefully considered when interpreting WAT stress responses in HFpEF. Although the results are intriguing, more studies are needed to explore this in detail.

Interestingly, paired analysis of adipose biopsies from HFpEF patients during acute decompensation and 1-month postdischarge revealed no statistically significant differences in transcript levels of browning markers (*UCP1*, *CIDEA*, *EVA*), white fat markers (*ADIPOQ*, *LEP*, *RETN*), inflammatory markers (*IL1B*, *IL6*, *TNFA*), fibrosis markers (*COL1A*, *COL3A*, *COL6A*), or endothelial markers (*CDH5*, *PECAM1*, *VEGFA*, *HIF1A*). These findings suggest that the acutely decompensated state does not induce a heightened adipose tissue stress response compared with the stable HFpEF state.

HFpEF remains an area of significant unmet clinical need, with limited pharmacological treatments currently available. By examining WAT in the context of HFpEF, Valero-Muñoz et al^5^ have offered important insights into adipose tissue remodeling, which may reflect an HFpEF-associated stress response and potentially inform future therapeutic strategies. The observation of WAT browning in HFpEF mice compared with obese sham control subjects is particularly compelling. It remains to be seen, however, whether the changes observed in mice are also fully validated in patients with HFpEF. Although there are indications of WAT alterations in these patients, the contribution of HFpEF itself, as opposed to confounding comorbidities, cannot yet be clearly delineated because of the correlational nature of clinical research. Furthermore, no significant differences in WAT markers were observed between the acutely decompensated and stable HFpEF states. Additional studies are needed to better understand these findings and to determine whether targeting adipose tissue could represent a viable therapeutic avenue in HFpEF, targeting critical adipocyte-derived factors, such as adipokines, metabolites and critical signaling lipids.
